# Cyr61 and YB-1 are novel interacting partners of uPAR and elevate the malignancy of triple-negative breast cancer

**DOI:** 10.18632/oncotarget.9853

**Published:** 2016-06-06

**Authors:** Michaela C. Huber, Natalie Falkenberg, Stefanie M. Hauck, Markus Priller, Herbert Braselmann, Annette Feuchtinger, Axel Walch, Manfred Schmitt, Michaela Aubele

**Affiliations:** ^1^ Institute of Pathology, Helmholtz Zentrum München, German Research Center for Environmental Health, Neuherberg 85764, Germany; ^2^ Research Unit of Protein Science, Helmholtz Zentrum München, German Research Center for Environmental Health, Neuherberg 85764, Germany; ^3^ Research Unit of Radiation Cytogenetics, Helmholtz Zentrum München, German Research Center for Environmental Health, Neuherberg 85764, Germany; ^4^ Research Unit of Analytical Pathology, Helmholtz Zentrum München, German Research Center for Environmental Health, Neuherberg 85764, Germany; ^5^ Clinical Research Unit, Department of Obstetrics and Gynecology, Technische Universität München, München 81675, Germany

**Keywords:** TNBC, YBX1, CCN1, proximity ligation assay, PLA

## Abstract

The triple-negative breast cancer (TNBC) is a very aggressive tumor type often occurring in young women and is associated with a bad prognosis for the patients. TNBC lacks established targets for breast cancer therapy, such as the estrogen receptor (ER), progesterone receptor (PR) and the human epidermal growth factor receptor 2 (HER2). Therefore, novel therapeutic targets and strategies are needed for an improved treatment of this breast cancer subtype. TNBC and respective cell lines often overexpress proteins of the urokinase plasminogen activator system (uPAS) including uPA, its receptor uPAR and inhibitor PAI-1, which together with co-factors contribute to the malignancy of TNBC. Here, two novel interacting partners of uPAR, the cysteine-rich angiogenic inducer 61 (Cyr61) and the Y-box-binding protein 1 (YB-1) were identified and their differential expression demonstrated in TNBC cells as well as in tumors. In the TNBC cohort, both interactors significantly correlated with expression levels of cathepsin B, c-Met and the tumor grade. In addition, expression levels of Cyr61 significantly correlated with cathepsin D (p=0.03), insulin receptor (p≤0.001), insulin-like growth factor receptor 1 (IGF1R, p=0.015) and also with YB-1 (p=0.0004) levels. The interactions of uPAR with Cyr61 significantly correlated with expression levels of tumor-promoting biomarkers including plasminogen (p=0.0014), cathepsin B (p=0.032), c-Met (p=0.0192) as well as with the tumor grade (p=0.02). In multivariate survival analysis, YB-1 showed independent prognostic value (p=0.01). As the novel interacting partners, also together with uPAR, contribute to tumor progression and metastasis, both may be potential therapeutic targets in breast cancer.

## INTRODUCTION

TNBC occurs in approximately 15% to 20% of breast cancers worldwide, mostly in young women and is clinically defined by the lack of expression of the receptors for estrogen and progesterone (ER/PR) and the absent overexpression of the human epidermal growth factor receptor 2 (HER2) [1]. Due to the absence of these therapeutic targets in breast cancer, the treatment of TNBC patients is limited to (neo) adjuvant chemo- and/or radiotherapy that is associated with severe side effects. Obviously, novel therapeutic approaches and targets are necessary. The molecular and physiological mechanisms of this heterogeneous breast cancer subgroup are not completely understood [2]. However, the most TNBCs and respective cell lines overexpress the major components of the urokinase-type plasminogen activator system (uPAS): uPA, its receptor (uPAR) and its inhibitor 1 (PAI-1) [3–6]. The uPAS plays an important role in cell movement during natural processes including wound healing, clot lysis and tissue remodeling leading to activation of further components and degradation of the extracellular matrix. In cancer, it is associated with enhanced migration and invasion of tumor cells and with a bad prognosis for the patients [7]. The uPAS components with interacting proteins and co-factors are interesting candidates as novel molecular targets or prognostic/predictive biomarkers for a tailored and improved therapy of TNBC. Regarding the clinical relevance of uPAS components, elevated uPA/PAI-1 levels have been shown being of prognostic and predictive value in breast cancer [8, 9]. Knockdown of uPAR was investigated in *in vitro* and *in vivo* studies, also in combination with downregulation of several tumor-promoting markers or trastuzumab, resulting in decreased tumorigenesis [10–12]. As uPAR is a membrane-associated and not a transmembrane receptor, it has to cooperate with interacting or associated partners, such as uPA, IGF1R, epidermal growth factor receptor (EGFR), integrins and vitronectin [13–17], to mediate intracellular signaling[11, 18]. The scope of the present study was to identify novel interacting partners of uPAR that may be potential therapeutic targets or of clinical relevance. Therefore, precipitates derived from uPAR-co-immunoprecipitations using the TNBC cell line MDA-MB-231 were subjected to mass spectrometric analysis followed by further supportive techniques. The cysteine-rich angiogenic inducer 61 (Cyr61) and the Y-box-binding protein 1 (YB-1) have been identified being novel interacting partners of uPAR. Based on immunohistochemical and clinical data of the patients, Cyr61 and YB-1 have been shown to be of clinical relevance in breast cancer including TNBC and may be promising therapeutic target.

## RESULTS

### Identification of Cyr61 and YB-1 as potential interaction partners of uPAR in MDA-MB-231 cells

To identify new interaction partners of uPAR, a co-immunoprecipitation (co-IP) was performed and analyzed by using liquid chromatography - tandem mass spectrometry (LC-MS/MS) on a LTQ Orbitrap XL mass spectrometer. The mass spectrometric analysis of the uPAR precipitate showed that in total, 258 proteins were detected with at least one unique peptide. Out of those, 106 proteins were enriched in comparison to the negative control precipitate and significantly enriched proteins (p≤0.05) are listed in Table 1. Based on ANOVA analysis, uPAR was on top of the protein list, as significantly enriched (21.1-fold, p≤0.001), which was expected and supported the significance of the results (Table 1; *PLAUR*). The already known direct interaction partners uPA (18.2-fold, p=0.003; *PLAU*), PAI-1 (9.1-fold, p=0.003; *SERPINE1*) and vitronectin (6.5-fold, p=0.05; *VTN*) were significantly enriched in the uPAR precipitate (Table 1). Among the significantly enriched proteins in the uPAR precipitate, the cysteine-rich angiogenic inducer Cyr61 (19.9-fold, p=0013; red in bold) and the Y-box-binding protein YB-1 (2.1-fold, p=0.002, red in bold) were selected as highly interesting candidates for being direct interaction partners of uPAR (Table 1). The selection of the novel interactors was based on the p-value, fold change and potential tumor-promoting relevance.

**Table 1:** List of selected known and potential interactors of uPAR identified by MS analysis

Protein IDs	Unique peptides	Anova (p-value)	Fold change co-IP/NC	Gene name	Name of (potential) interactor
^*^339328	13	0.000	21.1	*PLAUR*	plasminogen activator, urokinase receptor
^*^345893	3	0.000	69.3	*TJP2*	tight junction protein 2
^*^248437	1	0.001	4.8	*TUBA4A*	tubulin, alpha 4a
^*^361626	2	0.002	**2.1**	***YBX1***	Y-box-binding protein 1
^*^365625	4	0.003	68.4	*DHX16*	DEAH (Asp-Glu-Ala-His) box polypeptide 16
^*^361848	1	0.003	18.2	*PLAU*	plasminogen activator, urokinase
^*^223095	3	0.003	9.1	*SERPINE1*	serpin peptidase inhibitor (plasminogen activator inhibitor type 1)
^*^398736	1	0.013	**19.9**	***CYR61***	cysteine-rich angiogenic inducer 61
^*^339095	1	0.018	2.3	*RPS7*	ribosomal protein S7
^*^350170	1	0.026	14.8	*FXR1*	fragile X mental retardation, autosomal homolog 1
^*^346050	1	0.029	2.4	*RPS3A*	ribosomal protein S3A
^*^340329	4	0.029	1.4	*CAPRIN1*	cell cycle associated protein 1
^*^188376	1	0.050	5.0	*SLC25A3*	solute carrier family 25 (mitochondrial carrier; phosphate carrier), member 3
^*^226218	1	0.050	6.5	*VTN*	vitronectin

* ENSP00000

### Cyr61 and YB-1 are differentially expressed in breast cancer cell lines

**Figure 1:**
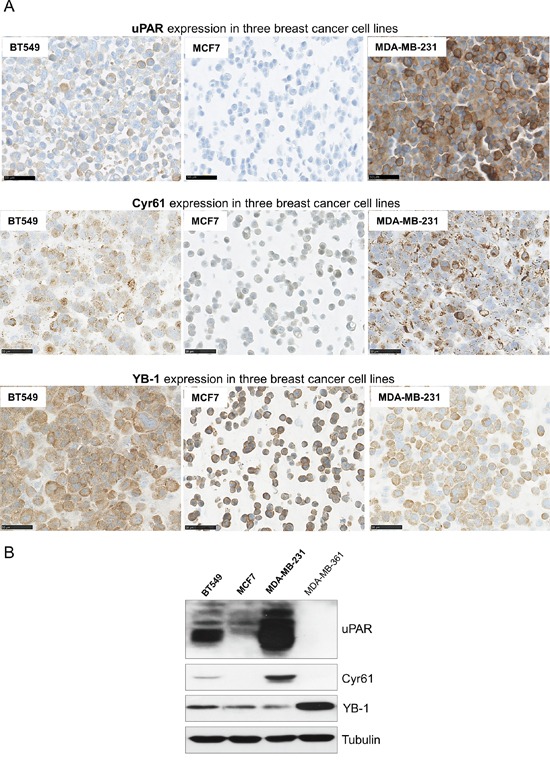
Differential expressions of uPAR, Cyr61 and YB-1 in breast cancer cell lines **A.** Immunohistochemical analysis of uPAR, Cyr61 or of YB-1 in the breast cancer cell lines BT549, MCF7 and MDA-MB-231, bar: 50 μm. **B.** Representative Western blot analysis of uPAR, Cyr61 and YB-1 in BT549, MCF7, MDA-MB-231 and MDA-MB-361 breast cancer cell lines. Tubulin was used as loading control.

To determine if the new identified potential interaction partners are co-expressed with uPAR in different breast cancer cell lines, immunohistochemical as well as Western blot analyses were conducted. uPAR was detected in the TNBC cell lines BT549 and even stronger expressed in MDA-MD-231 cells, whereas it was not detectable in the MCF7 cells (Figure 1A). The IHC analysis revealed a membranous and cytoplasmic expression of Cyr61, which was strongly detectable in the MDA-MB-231 and in the BT549 cells in contrast to the rather low expression in the MCF7 cells (Figure 1A). YB-1 was detected in the cytoplasm and was differentially expressed in the analyzed cell lines (Figure 1A). Those results were supported by the Western blot analyses. In particular, the protein expression of Cyr61 was strong in the endogenously uPAR-overexpressing cell line MDA-MB-231 (Figure 1B). The TNBC cell line BT549 showed a protein expression of Cyr61, though not as strong as in the MDA-MB-231 cell line (Figure 1B). The other tested breast cancer cell lines (MCF7 and MDA-MB-361) did not show any Cyr61 expression, which is accompanied by a low or undetectable expression of uPAR in these cells and may indicate an uPAR-dependent expression.

The other identified potential interaction partner YB-1 could be detected in all used cell lines, however, the MDA-MB-231 and the MCF7 cells showed the lowest expression, followed by a higher expression in the BT549 cells and a strong expression in the MDA-MB-361 cells (Figure 1B).

### uPAR directly interacts with Cyr61 and with YB-1 in the MDA-MB-231 cells

**Figure 2:**
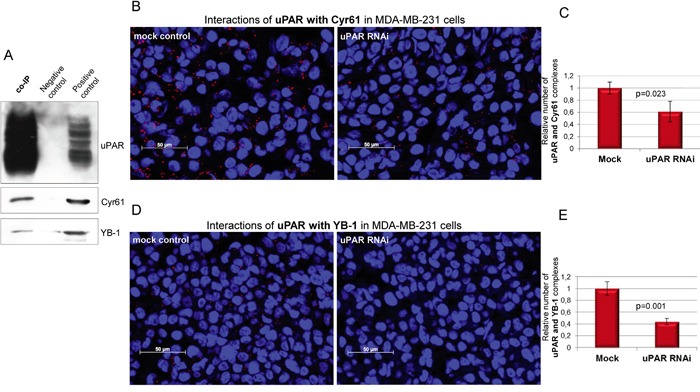
Cyr61 and YB-1 are novel and direct interacting partners of uPAR in MDA-MB-231 cells **A.** Detection of the novel interacting partners Cyr61 and YB-1 in the co-immunoprecipitates (co-IP) of uPAR. The polyclonal goat isotype antibody was used as negative control and MDA-MB-231 cell lysates as positive control. **B.** PLA analysis of the interactions of uPAR with Cyr61 and **C.** quantifications of respective PLA signals as well as **D.** of the interactions of uPAR with YB-1 and **E.** quantifications of respective PLA signals on FFPE cell block sections derived from mock control and uPAR-depleted (uPAR RNAi) cells; bar: 50 μm. Standard deviations and p-values are shown.

To support the results obtained with mass spectrometry analysis, the co-IP precipitates were examined by Western blots. The results clearly showed that beside uPAR, the novel direct interaction partners Cyr61 and YB-1 were enriched, whereas these proteins were not detected in the negative control (Figure 2A). The MDA-MB-231 cell lysates were used as positive control for the detection of the respective proteins (Figure 2A). In order to support that Cyr61 and YB-1 are novel direct interaction partners of uPAR, the proximity ligation assay was performed on FFPE cell block sections, which had been produced out of mock control and uPAR-depleted MDA-MB-231 cells. The proximity ligation assay was conducted to visualize the interaction of uPAR with either Cyr61 or YB-1 based on a co-localization of target proteins in close proximity (30-40 nm). uPAR did interact with each of the new identified proteins (Figure 2B and 2D). The number of the complexes of uPAR formed with Cyr61 was significantly reduced (to 62%, p=0.023) in uPAR-depleted cells (uPAR RNAi) in comparison to the mock control cells (Figure 2B and 2C). The amount of uPAR-YB-1 interactions was also significantly reduced (to 45%, p=0.001) following the uPAR downregulation in comparison to the control cells (Figure 2D and 2E). To underpin the specificity of the uPAR-Cyr61 complexes, additional PLA analyses were conducted on cell block sections derived from BT549 cells (differentially expressing both interactors, Figure 1A and 1B) as well as of MCF7 cells (expressing both interactors at very weak levels, Figure 1A and 1B). The amount of uPAR-Cyr61 complexes in BT549 cells was significantly higher (p=0.003) comparing the amounts in MCF7 cells (Supplementary Figure S1A and S1B).

### Cyr61 and YB-1 are strongly involved in tumor progression of TNBC

**Figure 3:**
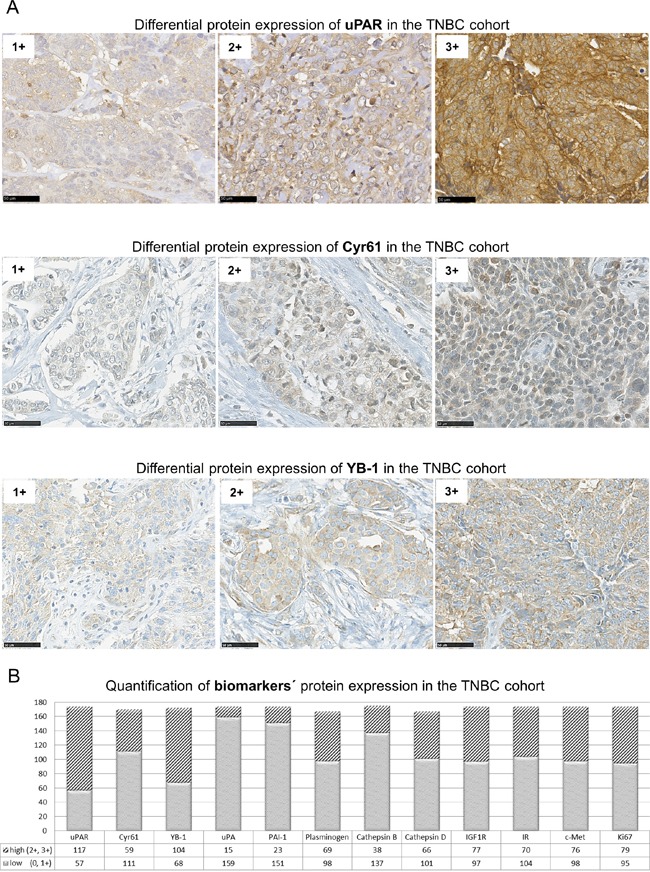
uPAR, Cyr61 and YB-1 are differentially expressed in TNBC **A.** Representative examples of immunohistochemical analysis of TNBC specimens showing differential expression (1+ to 3+) of uPAR, Cyr61 or of YB-1, bar: 50 μm. **B.** Quantification of biomarkers' protein expression with high (2+ and 3+) or low (0 and 1+) protein expression levels in the TNBC cohort (n≤174) determined by IHC analysis.

To assess the relevance of the new identified interaction partners of uPAR in the TNBC cohort (n≤174), tumor samples were immunohistochemically analyzed for the protein expression and localization of Cyr61 and YB-1. Both proteins were detectable in the cytoplasm and weakly at the cell membrane and were differentially expressed in the TNBC cohort (Figure 3A). Fifty-seven tumor samples showed low and 117 samples a high uPAR expression, 111 tumor specimens expressed Cyr61 in a low and 59 in a high level (Figure 3B). YB-1 was expressed low in 68 samples and strongly in 104 samples (Figure 3B). Following immunohistochemical analyses and quantifications of the novel interaction partners and of the other biomarkers in this TNBC cohort (Figure 3B), further statistical correlations were conducted. The Cyr61 expression significantly correlated with the expression of components of the uPA system including uPAR (p=0.04), uPA (p≤0.001, inverse), PAI-1 (p=0.002, inverse), plasminogen (p≤0.001), cathepsin B (p≤0.001) and cathepsin D (p=0.0302, Table 2). The Cyr61 expression significantly correlated with the expression of c-Met (p=0.0002), of IGF1R (p=0.0153), of the insulin receptor (p=0.0006), of YB-1 (p=0.0004) and with the histological grade of the tumors (p=0.01, Table 2).

**Table 2:** Statistically significant correlations of expressions of Cyr61 or of YB-1 with further biomarkers and clinical data

Interactor	Biomarker	p-value	Spearman's correlation coefficient
**Cyr61**	uPAR	=0.0399	0.17
	uPA	=0.0013	−0.25
	PAI-1	=0.002	−0.25
	Plasminogen	≤0.0001	0.32
	Cathepsin B	≤0.0001	0.32
	Cathepsin D	=0.0302	0.17
	IGF1R	=0.0153	0.19
	Insulin Receptor	=0.0006	0.26
	c-Met	=0.0002	0.28
	YB-1	=0.0004	0.27
	Histological grade	=0.01	0.20
**YB-1**	Cathepsin B	=0.0011	0.25
	c-Met	=0.0008	0.26
	Ki67	≤0.0001	0.47
	Histological grade	=0.002	0.25

In this TNBC cohort, the expression of YB-1 significantly correlated with the expression of cathepsin B (p=0.0011), c-Met (p=0.0008), Ki67 (p≤0.0001) and with the histological grade of the tumors (p=0.002, Table 2). In multivariate analyses for distant metastases-free survival of patients, YB-1 showed independent prognostic value (p=0.01) in addition to the lymph node status (p=0.002).

### Cyr61 and uPAR interaction significantly correlates with a malignant potential in TNBC

**Figure 4:**
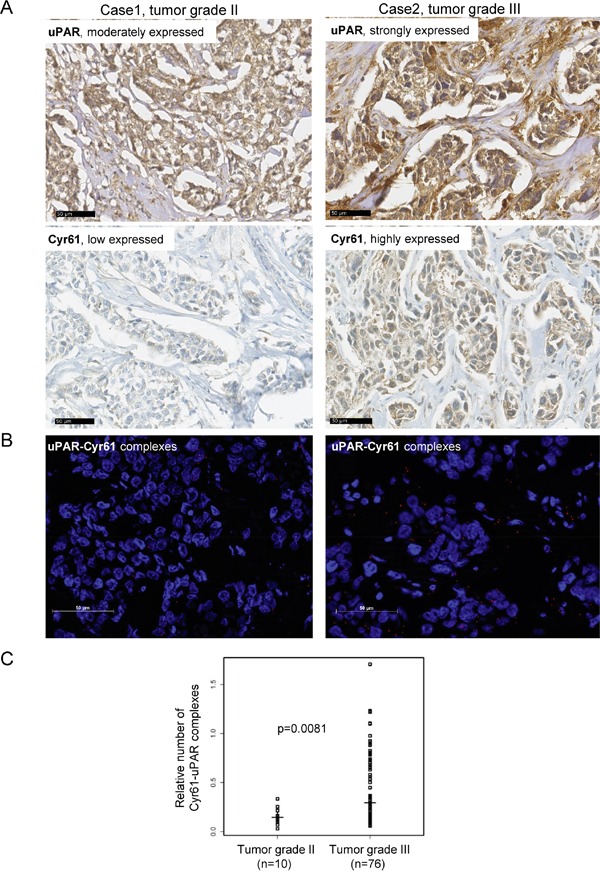
The uPAR and Cyr61 complexes correlate with the tumor grade in TNBC **A.** Representative examples of immunohistochemical analysis of uPAR and Cyr61 in specimens showing tumor grade II or tumor grade III, bar: 50 μm. **B.** Visualisation of uPAR-Cyr61 complexes in a tumor grade II or tumor grade III specimen, bar: 50 μm. **C.** For the statistical analysis of the uPAR-Cyr61 complexes (n=97) vs the tumor grade II (n=10) and grade III (n=76) the Wilcoxon rank test was conducted. The medians and the p-value are shown.

Since in this TNBC cohort, the Cyr61 expression significantly correlated with several tumor-promoting biomarkers, here, the clinical relevance of the new identified uPAR-Cyr61 interaction was analyzed in more detail by a proximity ligation assay. Apart from the expected correlations of these interactions with each of the interacting partners, the uPAR-Cyr61 complexes significantly correlated with plasminogen (p=0.0014), cathepsin B (p=0.032), c-Met (0.0192), uPA (0.0013, inverse), PAI-1 (p=0.008, inverse) and with the histological grade of the tumors (p=0.02, Table 3). The specimens, which differentially expressed uPAR and Cyr61 with either tumor grade 2 or 3 (Figure 4A) were further analyzed with regard to associations with tumor grading. Low amounts of the uPAR-Cyr61 complexes were detected in tumor samples with grade 2, whereas higher amounts of uPAR-Cyr61 complexes were mostly detected in samples with tumor grade 3 (Figure 4B) and the correlation was statistically significant (p=0.0081, Figure 4C).

**Table 3:** Statistically significant correlations of uPAR-Cyr61 protein complexes with further biomarkers and clinical data

Protein complex	Biomarker	p-value	Spearman's correlation coefficient
**uPAR-Cyr61**	uPA	=0.0013	−0.33
	PAI-1	=0.008	−0.28
	Plasminogen	=0.0014	0.34
	Cathepsin B	=0.032	0.22
	c-Met	=0.0192	0.25
	Histological grade	=0.02	0.25

## DISCUSSION

Since there is still a lack of targeted therapies in TNBC, the uPA system as well as novel interacting partners of uPAR or associated proteins are of great interest as candidates for therapeutic biomarkers in this aggressive tumor entity. To explore the uPA system as a potential therapeutic target system and associated proteins, here, Cyr61 and YB-1 were identified as novel interaction partners based on analyzing an uPAR-co-IP-precipitate using mass spectrometry. The protein expressions as well as the localization of both candidates were shown in appropriate cell lines and tumor samples. Interestingly, the expression of Cyr61 seemed to be associated with uPAR expression. The matricellular protein Cyr61, which *in vivo* is highly relevant for the induction of angiogenesis [19], one of the key factors for tumor growth and metastasis [20], binds to proteins in the extracellular matrix [21, 22] and is shown here being expressed in the TNBC cell lines MDA-MB-231 and BT549. A direct connection of Cyr61 with the uPA system was shown through Cyr61 cleavage by plasmin [23]. A siRNA-based transient knockdown and the antibody-based inhibition of Cyr61 in MDA-MB-231 cells led to a reduced cell invasion and migration [24]. In osteosarcoma, the silencing of Cyr61 led to a reduced vascularization *in vivo* [25]. Cyr61 binds to the somatomedin B^(1-44)^-domain of vitronectin [26], which is one of the already described direct interaction partners of uPAR [27]. Moreover, Francischetti and colleagues showed that after the incubation of immobilized vitronectin with Cyr61, the added uPAR-positive cells attached less frequently to vitronectin than in the untreated controls [26]. Cyr61 also bound to integrin αvβ3 [28], through which uPAR induced intracellular signaling [18].

Here, we precipitated Cyr61 with uPAR in the TNBC cell line MDA-MB-231. We also demonstrated complexes of these two proteins in tumor cells and tumor samples by PLA, which were significantly reduced following uPAR knockdown and underpinned a direct interaction of these proteins. However, it remains unclear if Cyr61 formed a complex with uPAR alone or in combination with the integrin αvβ3 or possibly vitronectin. Further analyses are needed to clarify this. The analyses of Cyr61 expression in our TNBC cohort revealed that about two third of the specimens were Cyr61-positive (1+ to 3+) and significantly correlated with uPAR expression levels and inversely with uPA and PAI-1 expression levels, which might be due to a common upstream regulation by for example plasmin. Also possible is that uPA and Cyr61 might be competitors for binding uPAR. In our study, the Cyr61 expression significantly correlated with the expression of the cathepsins B and D, which have been described to be associated with invasion and metastasis of cancer [29, 30]. Furthermore, a significant correlation of Cyr61 with the receptor tyrosine kinases insulin receptor (IR), insulin-like growth factor receptor (IGF1R) and c-Met was shown here. This is in agreement with a previous study showing that Cyr61 expression in MCF7 cells was significantly increased following stimulation with IGF-1 [31].

In our TNBC cohort, the Cyr61 expression also significantly correlated with the histological grade of tumors. This outcome supported the hypothesis that Cyr61 expression may be associated with a more aggressive phenotype. To date, Cyr61 expression was shown to significantly correlate with the histological grade in a tumor cohort of 61 DCIS (ductal carcinoma *in situ*) specimens, however, the analyzed samples were not specifically triple-negative [32]. Our present study revealed a statistically significant correlation of the tumor grade with Cyr61 expression and with the uPAR-Cyr61 complexes. In detail, we have demonstrated that higher number of uPAR-Cyr61 complexes significantly correlated with a higher tumor grade indicating higher metastatic potential of uPAR together with Cyr61 in TNBC.

In addition to Cyr61, YB-1 was identified as a new interaction partner of uPAR. YB-1, a member of the cold-shock protein family, seems to be part of the regulation of several signaling cascades affecting cellular proliferation, survival and invasion [33]. It is frequently expressed in various tumors including the non-small cell lung carcinoma [34] or the mammary carcinoma, where it might have prognostic value [35]. Its nuclear as well as cytoplasmic expression in breast cancer was previously reported [36, 37]. Depending on the specificity of the used antibody, YB-1 could be detected in the nucleus or in the cytoplasm. For this study, we have applied an antibody, which is directed against the carboxy terminus of YB-1 detecting its cytoplasmic expression [38]. With regard to its relevance for cellular invasion, YB-1 was also analyzed in connection to the uPA system. Downregulation of YB-1 led to an inhibition of uPA and overexpression of YB-1 led to an induced uPA expression [39]. In our present study, we showed that YB-1 was precipitated and formed protein complexes with uPAR, which were significantly reduced in uPAR-depleted cells. However, in our TNBC cohort, no correlation of YB-1 with the components of the uPAS was observed. This outcome is in agreement with a previous study [37] indicating that it is not always possible to directly compare the results derived from cell lines and tumors, in particular when the analyses were conducted with different techniques. Further analyses of the YB-1 expression in our TNBC cohort, revealed a significant correlation with the histological grade of the tumors. Wang and colleagues found no statistical association with the histological grade but an association of YB-1 expression and a bad prognosis for a 5-year-overall survival [35]. Such an association of YB-1 expression and bad prognosis for 5-year-survival was shown previously in a smaller cohort suggesting a prognostic and predictive role for YB-1 in breast cancer [35, 37]; however, the relevance of YB-1 for TNBC was not explicitly analyzed. We have shown YB-1 to be differentially expressed also in non-TNBC cell lines and its independent prognostic value in survival analysis. Thus, it could be possible that YB-1 is not especially relevant for TNBC but might be relevant for the malignancy as well as a potential therapeutic target in several breast cancer entities and needs further clarification.

Taken together, to the best of our knowledge, we are the first who have identified Cyr61 and YB-1 as new interaction partners of uPAR and showed that their expression correlated significantly with the expression of tumor-promoting biomarkers and the tumor grade in TNBC specimens. Our results point out the potential of uPA system and associated proteins, such as Cyr61 and YB-1 as promising and novel direct targets for a tailored therapy of breast cancer including TNBC. The generation of specific inhibitors e.g. small molecules or RNAi-based technologies targeting overexpressed uPAR-interactors, such as Cyr61 or YB-1, are thinkable as future anti-cancer treatments. Thus, the uPA system- or uPAR-mediated migration and invasion of tumor cells could be diminished. Considering the impact of Cyr61 also in angiogenesis, which is important for tumor progression [19], its inhibition may improve breast cancer therapy by impeding induction of angiogenesis.

## MATERIALS AND METHODS

### Cell culture and stable downregulation of uPAR

The human breast cancer cell line MDA-MB-361 (HTB-27) was purchased from American Type Culture Collection (ATCC) and the MCF7 cell line was obtained from the German Collection of Microorganisms and Cell Culture (DSMZ). The MDA-MB-231 cells and the BT549 cells were a kind gift by Prof. M. Schmitt (Clinical Research Unit, Department of Obstetrics and Gynecology, Technische Universität München). The DMEM GlutaMAX Medium (Dulbecco's Modified Eagle's Medium) (Life Technologies, Darmstadt, DE) was used for culturing MDA-MB-361 cells and for the MDA-MB-231 cell culture 1% MEM Non-essential amino acids (Life Technologies, Darmstadt, DE) were added. The MCF7 and the BT549 cells were maintained in RPMI 1640 GlutaMAX Medium (Roswell Park Memorial Institute Medium) (Life Technologies, Darmstadt, DE) supplemented with 10 μg/ml bovine insulin (Sigma, St. Louis, MO, USA). To both media 10% FCS (fetal calf serum) (Invitrogen, Carlsbad, CA, USA) and 0.25% of each penicillin and streptomycin (Life Technologies, Darmstadt, DE) were added. The cells were cultured at 37°C and 5% CO_2_. The last cell line authentication was conducted before starting the experiments as described previously [40].

For stably downregulating uPAR in the MDA-MB-231 cells, a combination of three SMARTchoice™ lentiviral shRNA vectors (GE Healthcare Lafayette, CO, USA) each with a multiplicity of infection (MOI) of 30, were used (VSH6063, SH-006388-01, -02, -03). All viral particles were tested for knockdown specificity and efficiency before starting the RNAi experiments. A total of 3.0 × 10^4^ MDA-MB-231 cells were seeded into each well of a 12-well plate and after 42 h infected with the lentiviral vectors for the knockdown of uPAR (uPAR RNAi). The infection was repeated based on the method described [41]. For enhancing the infection, 2 μg/ml polybrene (Invitrogen, Carlsbad, CA, USA) was added to each approach as described [40]. All infections were conducted in triplicates.

### Western blot analysis

Protein expression of uPAR [42], Cyr61, YB-1 and α-Tubulin were analyzed by Western Blot (the antibodies are listed in the Supplementary Table S1) as described previously [43].

### Co-immunoprecipitations

Based on the method described before [44], a co-immunoprecipitation for uPAR and interaction partners was established. The polyclonal anti-uPAR antibody (AF807, R&D Systems, Minneapolis, MN, USA) as well as the polyclonal goat isotype antibody (026202, Invitrogen, Carlsbad, CA, USA) as negative control was incubated for 60 min at 4°C in the lysate and afterwards incubated with A/G PLUS beads (sc2003, Santa Cruz Biotechnology, Heidelberg, DE) for 15 h at 4°C. The precipitated proteins were eluted with 2x Laemmli buffer and analyzed by mass spectrometry and Western blot.

### Mass spectrometry analysis

Laemmli eluates were proteolyzed with trypsin using a modified Filter Aided Sample Preparation (FASP) [45] protocol as described [46]. Resulting peptides were acidified (trifluoracetic acid to final pH 2) and directly used for analysis on a LTQ-OrbitrapXL connected with an Ultimate3000 nano HPLC system as described [46, 47]. The full-scan MS spectra were acquired in the Orbitrap with a resolution of 60.000 and up to 10 most abundant peptide ions were selected for fragmentation in the linear ion trap. Peptides were identified and quantified using the Progenesis QI software (Nonlinear, Waters) and the Mascot search algorithm with the Ensembl Human public database as described [48].

### Preparation of FFPE cell line blocks

For the analyses of protein-protein interactions and single protein expression, formalin-fixed and paraffin embedded (FFPE) blocks were generated out of the cell lines MDA-MB-231 (mock control and uPAR-depleted), BT549 and MCF7 as described [49].

### Patients and tumor specimens

Formalin-fixed, paraffin-embedded breast cancer tissues of the TNBC type from female patients (n≤174) were collected at the Department of Gynecology and Obstetrics, Klinikum Rechts der Isar, Technische Universität München, Germany. Written informed consent for the use of tissue samples for research purposes was obtained from all the patients and approval for the use of the tumor samples was given from the Ethics Committee of the Medical Faculty of the Technische Universität München, Germany. By immunohistochemical analysis, the low or negative expression of HER2, estrogen and progesterone receptor was verified. Ninety tumors were classified as node-negative, 52 tumors were less than 2 cm in size, 93 were between 2 to 5 cm and 29 tumors were larger than 5 cm. Most of the tumors (n=150) were classified as grade 3, 21 cases were grade 2 and 3 tumors were grade 1 [50]. All patients were treated with surgery and 108 patients received adjuvant chemotherapy. The median follow-up of the patients was 57 months (max. 244 months) and 30% of the patients (n=52) suffered from distant metastases within this time of clinical follow up.

### Generation of tissue microarrays (TMAs)

Using the tissue-arraying instrument (Beecher Instruments Inc., Silver Spring, MD, USA) TMAs were generated as described [51]. Three micrometers thick sections were cut from both, the TMA block and from the primary tumor blocks and Hematoxylin-Eosin stained to re-examine and validate representative sampling. The 3 μm thick TMA sections were cut for immunohistochemical and PLA analyses.

### Immunohistochemical (IHC) analysis

To determine the expression and localization of proteins in cells and tumor specimens, IHC analysis was performed using an automated stainer (Discovery XT, Ventana Medical Systems, Tucson, AZ, USA) with a DAB Map kit (Ventana Medical Systems, Tucson, AZ, USA) as described [51]. The used antibodies are listed in the Supplementary Table S1. The stained TMA samples were scored by two independent observers using a 4-point scale (0-3+) [51] and the cut-off for positivity was ≥1+.

### Proximity ligation assay (PLA)

To visualize and quantify the complex formation of uPAR with interactors, the PLA was performed on sections of cell line blocks and of the TMA blocks using the DUOLink™ kit (OLINK Bioscience, Uppsala, S) according to the manufacturer's instruction as described [40, 52]. The primary antibodies against uPAR (1:200) and Cyr61 (1:1200), which were used for IHC analysis (Supplementary Table S1), were applied. The cell lines MCF7 or BT549 showing no or low expression levels of the target proteins were used as controls. The PLA signals were detected using a confocal laser scanning microscope (AxioImager, Zeiss, Jena, DE) and three visual fields per sample were captured, followed by three-dimensional image projection and conversion to TIF format [52]. The PLA signals were analyzed using the Definiens software (Definiens Enterprise Image Intelligence Suite software, Munich, DE). The mean number of protein complexes was calculated per 1000 pixels of tissue area followed by statistical analysis of signal frequencies as described [52].

### Statistical analysis

For correlating the experimental parameters with the histopathological parameters (lymph node status, tumor size, histological grade) and the clinical course of the disease (age, local and distant metastases-free survival), the Spearman's rank correlation test or the Wilcoxon rank test were used. Results were considered as statistically significant when p≤0.05. In multivariate analysis, all parameters with univariate significance of at least p≤0.15 were offered to the analysis.

## SUPPLEMENTARY FIGURE AND TABLE


