# Supplementary material for: Cyr61 and YB-1 are novel interacting partners of uPAR and elevate the malignancy of triple-negative breast cancer
Source: Oncotarget. 2016 Jun 6;7(28):44062–75. doi: 10.18632/oncotarget.9853 (PMC5190079; doi:10.18632/oncotarget.9853)
Supplement: Supplementary file 1 [file oncotarget-07-44062-s001.pdf]

Cyr61 and YB-1 are novel interacting partners of uPAR and elevate the malignancy of triple-negative breast cancer

SUPPLEMENTARY FIGURE AND TABLE

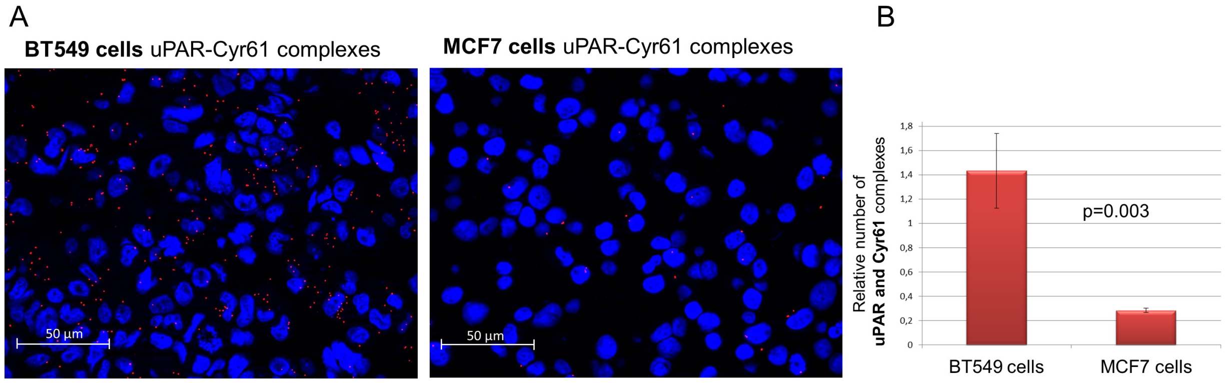

Supplementary Figure S1: uPAR and Cyr61 directly interact in breast cancer cell lines. A. PLA analysis of the interactions of uPAR with Cyr61 and **B.** quantifications of respective PLA signals on FFPE cell block sections derived from BT549 (differentially expressing the interactors) and MCF7 cells (weakly expressing the interactors); bar: 50 µm. Standard deviations and p-value are shown.

Supplementary Table S1: The list of optimized concentrations of antibodies used for immunohistochemical analyses (IHC) and/or Western blots (WB)

Antigen	Catalogue number and manufacturer	Application	
		IHC	WB
uPAR	IID7 [42]	1:500	1:500
Cyr61	sc13100, Santa Cruz Biotechn., Heidelberg, Germany	1:800	1:1000
YB-1	4202, Cell Signaling Technology, Beverly, MA, USA	1:200	1:1000
uPA	3689, American Diagnostica, Stamford, CT, USA	1:1000	
PAI-1	3786, American Diagnostica, Stamford, CT, USA	1:500	
Plasminogen	ab10178, Abcam Inc, Cambridge, MA, USA	1:35	
Cathepsin B	3373, Cell Signaling, Techn., Beverly, MA, USA	1:100	
Cathepsin D	PDR 004, Diagnostic BioSystems, Ontario, Canada	1:50	
IGF1R	3027, Cell Signaling, Techn., Beverly, MA, USA	1:50	
IR	HPA036302, Sigma Aldrich, St. Louis, MO, USA	1:15	
c-Met	ab51067, Abcam Inc, Cambridge, MA, USA	1:500	
Ki67	ab15580, Abcam Inc, Cambridge, MA, USA	1:1000	
Tubulin	T5168, Sigma Aldrich, St. Louis, MO, USA		1:5000
